# Long-term results of augmented unilateral lateral rectus muscle recession for dissociated horizontal deviation

**DOI:** 10.1371/journal.pone.0234017

**Published:** 2020-06-01

**Authors:** In Jeong Lyu, Jong Chul Han, Sei Yeul Oh

**Affiliations:** 1 Department of Ophthalmology, Korea Cancer Center Hospital, Korea Institute of Radiological and Medical Sciences, Seoul, Republic of Korea; 2 Department of Ophthalmology, Samsung Medical Center, Sungkyunkwan University School of Medicine, Seoul, Korea; Faculty of Medicine, Cairo University, EGYPT

## Abstract

We report the long-term surgical results of augmented lateral rectus muscle (LR) recession for dissociated horizontal deviation (DHD) without concomitant exotropia (XT) or esotropia (ET). This retrospective review included patients with DHD without XT or ET who underwent augmented LR recession and were followed-up for ≥12 months. Each patient’s medical records were evaluated to identify their demographics, preoperative angle of deviation, surgical procedure, success rate, and reoperation rate. A total of 11 patients with DHD were included (median patient age at surgery, 6 years; interquartile range [IQR], 5 to 10 years). Four patients (36.4%) had a history of infantile ET while three patients (27.3%) had a history of unilateral cataract surgery for congenital cataract. The median preoperative angle of DHD in the unilateral eye was 20 prism diopters (PD) (IQR, 15 to 25 PD). The median amount of LR recession was 8.0 mm (IQR, 7.5 to 8.0 mm). Three patients (27.3%) underwent simultaneous surgery for dissociated vertical deviation. At the final examination after a median follow-up period of 32 months (IQR, 24 to 58 months), 10 patients (91%) showed successful alignment. The long-term successful alignment rate after augmented LR recession for DHD was good; thus, application of this technique is appropriate in patients with DHD.

## Introduction

Dissociated strabismus complex (DSC) is a slow, disconjugate ocular deviation that elevates, abducts, and excyclotorts the eye.[1] It is usually bilateral and asymmetric. DSC occurs when normal binocular visual development is disrupted by infantile esotropia (ET) or exotropia (XT), congenital cataract, congenital optic nerve or retinal disease, and amblyopia.[2–4] The most common subtype of DSC is dissociated vertical deviation (DVD), which is characterized by slow elevation of the non-fixating eye.[5] In contrast to DVD, dissociated horizontal deviation (DHD) is a less well-known manifestation of DSC and is diagnosed when the horizontal component of DSC is exaggerated. It can be distinguished from intermittent XT by its asymmetry of the exodeviation between two eyes, and association with vertical and torsional movements.[1]

In 1991, Wilson and McClatchey[1] reported the findings for six patients with DHD who underwent lateral rectus muscle (LR) recession with 5 to 27 months of postoperative follow-up. In 2000, Wilson et al.[6] recommended 5 and 7 mm of unilateral LR muscle recession for unilateral DHD under and over 15 PD, respectively. However, the surgical outcomes for DHD, especially DHD without concomitant XT or ET, remain underreported.

Thus, the aim of this study was to present cases of DHD without concomitant ET or XT and their long-term surgical outcomes after augmented graded LR recession.

## Methods

We retrospectively evaluated the records of patients aged <20 years who underwent unilateral LR recession for DHD without concomitant ET or XT between 2010 and 2017 at Samsung Medical Center. Consecutive patients who were followed-up for >12 months postoperatively were included in this study. Patients with systemic disorders, neurologic disorders, bilateral severe amblyopia, or a prior history of LR surgery were excluded from this study. However, patients with a history of infantile ET, congenital cataract, or monocular intraocular anomaly were not excluded because of the pathogenesis of DSC. This study was approved by the institutional review board of Samsung Medical Center (Seoul, Republic of Korea) and conducted according to the Declaration of Helsinki.

Comprehensive ophthalmic examinations were performed in all patients. Ocular alignment was tested by cover/uncover and prism alternate cover testing at 6 m and 33 cm fixation. The prism undercover test is performed by first estimating the size of the DHD. Then, a prism equal to the estimated size of the deviation was placed base in in front of the affected eye, while the eye was dissociated behind a translucent occluder and then rapidly switched to the contralateral eye. We confirmed the angle of deviation with no adducting movements of the eye, taking into account the amplitude of the underlying latent nystagmus. In cases with monocular visual acuity of <20/200, the Krimsky test was performed for measurement of the angle of deviation. We distinguished DHD from intermittent XT on the basis of the following factors: unilateral or asymmetrical amplitude of exodeviation dependent on the fixating eye, slow spontaneous exodeviation, variable amplitude of spontaneous exodeviation, the presence of Bielschowsky phenomenon, and associated latent nystagmus or prominent dissociated vertical divergence.[7] In addition, a reverse fixation test was also performed to define the dissociated component.[8, 9] DVD was diagnosed when the following findings were observed: an alternating hyperdeviation of the occluded eye or a hyperdeviation of one eye without a corresponding hypodeviation of the contralateral eye that violates Haring’s law. DVD is usually comitant, with the same measurements in all positions of gaze. When the size of the vertical deviation changed in the abduction, adduction, primary, and head tilt positions, we carefully examined the coexistence of abnormal inferior oblique (IO) or superior oblique (SO) function that caused incomitant DVD.

The following information was recorded: age at surgery, gender, best-corrected visual acuity (BCVA), previous surgical history, underlying ocular problems, concomitant DVD or other vertical deviations, and latent nystagmus. In addition, the preoperative amount of ocular misalignment, amount of LR recession, final ocular alignment, and follow-up period were also recorded.

One author (SYO) performed postoperative ophthalmic examinations and surgical procedures. We applied a new augmented dosage scheme for LR recession instead of the previously suggested dosage recommendations for DHD.[6, 10] We generally performed ipsilateral LR recession of 6.0 mm for 12 prism diopters (PD), 7.0 mm for 15 PD, 8.0 mm for 20 PD, 8.5 mm for 25 PD, and 8.0 mm with posterior fixation suture for DHD with exodeviation of ≥30 PD. The amount of LR recession was determined based on the maximal angle of DHD at a distance. The surgical target was orthotropia.

Postoperative horizontal alignment was considered satisfactory if the alignment was between 8 PD of exodeviation and 5 PD of esodeviation. Recurrence was defined as >8 PD of exodeviation and overcorrection was defined as >5 PD of esodeviation. Superior rectus muscle (SR) recession or IO anteriorization for DVD was undertaken simultaneously if the concomitantly manifesting DVD was large and occurred frequently. SO strengthening or IO weakening procedures were performed simultaneously if patients showed vertical misalignment in the primary position with abnormal head posture due to SO palsy or primary IO overaction. Modification of the surgical doses of LR recession was not performed, regardless of concurrent SR, IO, or SO surgery.

## Results

### Underlying ocular problems

We identified 92 patients with DHD during the study period. Among these, 23 patients had undergone surgery for DHD without exotropia/esotropia. Nineteen of the 23 patients were followed-up for more than 12 months. Among these, eight patients who received medial rectus (MR) resection, MR advancement, or LR re-recession were excluded. Finally, 11 patients who underwent LR recession for DHD without ET/XT and were followed-up for ≥12 months postoperatively were included in the study.

The demographics and surgical results of the patients are presented in Table 1. Median follow-up duration was 32 months (interquartile range [IQR], 24 to 58 months) from the operation for DHD. Four patients were male and seven were female. The median age of these patients at surgery was 6 years (IQR, 5 to 10 years). Four patients (36.4%) had a history of infantile ET, which was corrected by bilateral MR recession previously. Three patients (27.3%) had a history of unilateral cataract surgery for congenital cataract. One patient (9.0%) had a history of unilateral MR recession for a partially accommodative ET. In addition, one patient (9.0%) had a history of chorioretinitis and one patient (9.0%) had amblyopia. Lastly, one patient (9.0%) had no predisposing factor. Latent or manifested latent nystagmus was seen in six (54.5%) patients. Seven patients (63.6%) showed concomitant DVD, and among these patients, three (27.3%) underwent simultaneous surgery for DVD, which was large and occurring frequently; two patients had coexisting DVD without IO overaction and underwent SR recession. One patient with coexisting DVD and IO overaction underwent IO anteriorization. Another patient (9.0%) who showed concomitant secondary IO overaction with SO palsy without DVD underwent simultaneous IO recession.

**Table 1:** Clinical summary of 11 patients with dissociated horizontal deviation who underwent augmented unilateral lateral rectus muscle recession.

N	Age	Sex	BCVA		Previous history	Preoperative angle of DHD	Coexisting DVD	Nystagmus	Surgical procedure	Follow-up, months	Postoperative angle
			OD	OS							
1	5	M	20/25	20/25	infantile ET s/p B) MR recession	L) 20 PD	B) ≤5 PD	Yes	L) LR recession (8.0 mm)	31	No DHD
											B) ≤5 PD DVD
2	6	F	20/25	20/32	infantile ET s/p B) MR recession	L) 15 PD	B) ≤5 PD	Yes	L) LR recession (8.0 mm)	12	4 PD ET
											B) ≤5 PD DVD
3	5	F	20/50	20/50	infantile ET s/p B) MR recession s/p L) IO recession s/p L) SR recession d/t L) DVD,	L) 20 PD	R) 20 PD	Yes	L) LR recession (7.5 mm)	32	6 PD XT
									R) SR recession (5.0 mm)		No DVD
4	4	F	20/25	20/25	IET s/p B) MR recession	L) 12 PD	B) 15 PD	None	L) LR recession (5.5 mm)	36	2 PD ET
									B) IO anteriorization		No DVD
5	12	F	20/20	CF	L) congenital cataract, s/p cataract surgery	L) 20 PD	L) 20 PD	Yes	L) LR recession (8.0 mm)	60	No DHD
									L) SR recession (6.5 mm)		L)10 PD DVD
6	8	F	CF	20/20	R) congenital cataract, s/p cataract surgery	R) 15 PD	R) ≤5 PD	None	R) LR recession (8.0 mm)	26	4 PD ET
											R) ≤5 PD DVD
7	15	F	20/20	20/400	L) congenital cataract, s/p cataract surgery	L) 20 PD	None	Yes	L) LR recession (8.0 mm)	22	No DHD
8	5	F	20/20	CF	L) chorioretinitis	L) 35 PD	L) ≤5 PD	Yes	L) LR recession (8.0 mm), posterior fixation suture	135	No DHD
											No DVD
9	5	M	20/20	20/20	None	L) 25 PD	None	None	L) LR recession (8.0 mm)	56	L) 15 PD DHD
10	6	M	20/40	20/50	B) amblyopia	L) 12 PD	L) 6 PD HT d/t SOP	None	L) LR recession (6.0 mm)	81	4 PD XT
									L) IO recession (Grade II)		
11	19	M	20/20	20/25	partially accommodative ET, amblyopia s/p L) MR recession	L) 25 PD	None	None	L) LR recession (8.5 mm)	14	L) 8 PD DHD

BCVA = best-corrected visual acuity; DHD = dissociated horizontal deviation; DVD = dissociated vertical deviation; ET = esotropia; s/p = status, postoperative; d/t = due to; CF = counting fingers; MR = medial rectus muscle; IO = inferior oblique muscle; SR = superior rectus muscle; IXT = intermittent exotropia; HT = hypertropia; SOP = superior oblique palsy

### Surgical outcomes

The median amount of DHD before operation was 20 PD (IQR, 15 to 25 PD) in the unilateral eye. The median amount of unilateral LR recession was 8 mm (IQR, 7.5 to 8.0 mm). None of our cases involved bilateral DHD.

At the final examination after a median follow-up period of 32 months (IQR, 24 to 58 months), 10 patients showed successful alignment. One patient (Case 9) who showed a DHD recurrence of 15 PD in the same eye underwent reoperation with 6.5 mm of MR resection at 56 months postoperatively, and then achieved 2 PD of XT at the final follow-up after another 16 months.

## Discussion

The clinical characteristics of DHD have been reported previously. DHD is usually related to the horizontal deviation associated with DVD in patients with an early-onset strabismus history.[11] DHD is a horizontal variable deviation that changes with fixation of each eye and is unrelated to weakness, restriction, or different accommodation.[11] Asymmetry is an important clinical characteristic of DHD. Wilson[1] reported that DHD may be very difficult to distinguish from intermittent XT without unilaterality or asymmetry of the exodeviation between the eyes. Thus, variability and difficulty with a clear endpoint during prism neutralization indicate the possibility of DHD. Accompanying nystagmus has also been reported as a clinical symptom of DHD.[1] Zubcov[12] noted that variations in horizontal strabismus when fixation changed from one eye to the other, secondary to an asymmetric nystagmus blockage syndrome, were referred to as DHD. About half of the patients had accompanying latent or manifested latent nystagmus in our study.

Infantile ET is one of the causes of DHD. Brodsky[8] reported that half of consecutive patients with XT after surgery for infantile ET had DHD. David[10] reported that 34 (7%) of 484 patients who underwent surgery for infantile ET exhibited DHD. Similar to a previous study, the most common predisposing factor for DHD in our cases was infantile ET (36.4%), followed by unilateral congenital cataract (27.3%). In addition, there was also a case with no predisposing factors.

Despite its ability to be disfiguring, DSC is often small and latent. In such cases, surgical correction of DSC is not needed. However, surgical correction for horizontal deviation should be considered if DHD is definite and manifested, irrespective of the surgical correction of DVD, which has been known to be ineffective for DHD.[10] LR recession is known as the treatment of choice for DHD; however, few studies have discussed the surgical outcomes and dosage recommendations for DHD. In 1991, Wilson et al. reported six cases of surgically corrected DHD[1] and in 2000, suggested surgical recommendations for DHD.[6] The former study analyzed 32 patients, including 16 patients showing DHD with concomitant XT/ET and 16 patients showing pure DHD without concomitant ET/XT. In the literature, five types of surgeries have been performed for DHD, including unilateral and bilateral LR recession with or without posterior fixation, and MR resection/advancement, in consideration of histories such as previous surgery and coincident strabismus.[6] Wilson et al. performed ipsilateral LR recession of 5 mm for 20 PD, 6 mm for 25 PD, and 7 mm for 30 PD DHD, and showed a 21.9% reoperation rate. Thus, they recommended 7 mm of unilateral LR muscle recession for unilateral DHD over 15 PD and 5 mm of unilateral LR muscle recession for unilateral DHD under 15 PD.[6] Another study by David et al.[10] reported that the surgical response was about 2.5 PD per mm of LR recession in patients with DHD, which was almost equal to or slightly greater than the values in the conventional horizontal surgical table. They stated that there was no significant difference between patients undergoing surgery for DHD without XT and those with coexisting XT. The previously mentioned DHD studies evaluated their patients with pure DHD and DHD with coexisting XT or ET together.[6, 10]

In our study, we presented the surgical outcome of pure DHD cases in which augmented graded LR recession was performed according to the maximal deviation of DHD. Overall, we generally performed ipsilateral LR recession of 6.0 mm for 12 PD, 7.0 mm for 15 PD, 8.0 mm for 20 PD, 8.5 mm for 25 PD, and 8.0 mm with posterior fixation suture for DHD with exodeviation of ≥30 PD (Table 2).

**Table 2:** Surgical dosages for unilateral lateral rectus muscle recession in patients with exodeviated dissociated horizontal deviation.

Angle of deviation, prism diopters	Amount of recession, mm	Number of eyes
12	6.0	2
15	7.0	2
20	8.0	4
25	8.5	2
30–35	8.0 with posterior fixation sutures	1

Among these 11 cases, 10 retained good eye alignment during the follow-up period. Only one case required reoperation due to recurrent DHD, and there was no significant consecutive ET. We performed a larger amount of LR recession compared to the surgical recommendation by Wilson et al.[6] Notably, this surgical table was similar to the surgical dosages used for unilateral rectus muscle recession in children with basic-type intermittent XT under 25 PD of angle of exodeviation.[13] The difference was that we added posterior fixation sutures in patients with DHD and monocular exodeviation of ≥30 PD. On the basis of our long-term surgical results, we suggest the augmented surgical table for patients with DHD.

There were several limitations to our study. First, this study was retrospective in design. Second, the sample size was small due to the relatively uncommon occurrence of pure DHD. Third, the follow-up intervals were not standardized. Prospective well-controlled studies with larger sample sizes are required to confirm our results.

In conclusion, although a variable angle is the nature of DHD, careful measurement of deviation angles and application of augmented graded LR recession based on the maximal angle of deviation for patients with DHD shows good long-term successful alignment rates.
